# Covalent Functionalization of Layered Double Hydroxides to Generate Peptide-Based SARS-CoV-2 Nanovaccine

**DOI:** 10.3390/ma18112449

**Published:** 2025-05-23

**Authors:** Alejandra E. Liñán-González, Sayma A. Rodríguez-Montelongo, Mariano J. García-Soto, Daniela Gómez-Zarandona, Susan Farfán-Castro, Gabriela Palestino, Raúl Ocampo-Pérez, Erika Padilla-Ortega, Omar González-Ortega, Sergio Rosales-Mendoza

**Affiliations:** 1 Centro de Investigación y Estudios de Posgrado, Facultad de Ciencias Químicas, Universidad Autónoma de San Luis Potosí, Av. Manuel Nava 6, Zona Universitaria, San Luis Potosí 78210, Mexico; a182334@alumnos.uaslp.mx (A.E.L.-G.); say.mont07@gmail.com (S.A.R.-M.); mariano.soto@uaslp.mx (M.J.G.-S.); a301145@alumnos.uaslp.mx (D.G.-Z.); palestinogabriela@uaslp.mx (G.P.); raul.ocampo@uaslp.mx (R.O.-P.); 2 Sección de Biotecnología, Centro de Investigación en Ciencias de la Salud Biomedicina, Universidad Autónoma de San Luis Potosí, Av. Sierra Leona 550, Lomas 2a Sección, San Luis Potosí 78210, Mexico

**Keywords:** layered double hydroxides, nanoclay, subunit nanovaccine, adjuvant, active conjugation

## Abstract

Nanoclays have gained attention in biological applications due to their biocompatibility, low toxicity, and cost-effectiveness. Layered double hydroxides (LDHs) are synthetic nanoclays that have been used as adjuvants and antigen carriers in nanovaccines developed through passive bioconjugation. However, performing active bioconjugation to bind antigens covalently and generate subunit nanovaccines remains unexplored. In this study, we investigated the synthesis, functionalization, and active conjugation of LDH nanoparticles to produce subunit nanovaccines with peptides from SARS-CoV-2. The synthesis of Mg-Al LDHs via a coprecipitation and hydrothermal treatment rendered monodisperse particles averaging 100 nm. Their functionalization with (3-aminopropyl)triethoxysilane was better than it was with other organosilanes. Glutaraldehyde was used as a linker to bind lysine as a model biomolecule to establish the best conditions for reductive amination. Finally, two peptides, P_2_ and P_5_ (epitopes of the SARS-CoV-2 spike protein), were bound on the surface of the LDH to produce two subunit vaccine candidates, reaching peptide concentrations of 125 and 270 µg/mL, respectively. The particles were characterized using DLS, TEM, XRD, TGA, DSC, and FTIR. The cytotoxicity studies revealed that the conjugate with P_2_ was non-toxic up to 250 µg/mL, while the immunogenicity studies showed that this conjugate induced similar IgG titers to those reached when aluminum hydroxide was used as an adjuvant.

## 1. Introduction

Vaccines against viruses based on subunits, i.e., the antigenic part of a pathogen, are created to avoid the use of live or attenuated viruses [1]. The antigenic parts can be proteins, polysaccharides, or peptides [2]. Among these, recombinant proteins and synthetic peptides are regularly reported in the production of subunit vaccines [3,4]. Moreover, there are some licensed recombinant subunit vaccines against viruses such as Hepatitis B, Human Papilloma, Influenza, and Herpes Zoster [5]. While subunit vaccines are safer than vaccines with live or attenuated viruses, they are generally less antigenic and require the use of adjuvants and booster shots [6]. In this regard, nanomaterials have been proposed as the adjuvants for subunit vaccines. These nanomaterials include gold nanoparticles, polymeric nanoparticles, silica nanoparticles, liposomes, and nanoclays [7]. The latter includes naturally occurring halloysite nanotubes, as well as synthetic layered double hydroxides (LDHs) and hectorite [8].

LDHs are inorganic nanomaterials that have garnered significant attention in recent decades due to their versatile properties, including ease of synthesis, biocompatibility, and biodegradability [9]. Commonly referred to as anionic clays, LDHs feature a hexagonal or octahedral crystalline structure. These materials are two-dimensional (2D), ionic, layered structures composed of positively charged metal hydroxide layers intercalated with exchangeable anions to maintain charge balance [10,11,12,13]. The brucite-like layers of Mg(OH)_2_ gain a positive charge through isomorphic substitution, where Mg^2+^ is replaced by Al^3+^. In hydrotalcite, carbonate ions serve as the primary intercalated anion [12]. The combination of multiple layers and interlayer spacing creates a stable structure reinforced by electrostatic interactions and hydrogen bonds. The nature of these interactions contributes to the biodegradability of LDHs under environmental or physiological conditions [9].

Previous studies have shown that LDHs are an ideal adjuvant for the delivery of proteins, peptides, and DNA antigens, as they stimulate both humoral and cellular immune responses. Such an effect makes them a promising tool for immunotherapy against cancers, infections, and viruses [14]. In 2010, Li et al. reported for the first time the use of LDHs as an adjuvant in a nanovaccine composed of LDH/DNA against melanoma. They demonstrated that this formulation could activate dendritic cells, generating a more effective antigen-specific immune response compared to those of DNA vaccines alone. This work laid the foundation for using LDHs in immunotherapy by showing that these complexes not only protect and transport genetic material, but also improve immune system activation [15].

Subsequently, various researchers developed nanovaccines targeting cancers, viruses, and bacteria, comparing their efficacy to those of commonly used adjuvants, such as Alum, Quil A, and Freund’s adjuvants. LDHs ranging in size from 56 to 215 nm have been tested with different antigens to immunize BALB/c and C57BL/6 mice, as well as pigs [16]. These studies included model antigens, such as OVA [17] and BSA [18], as well as DNA [19] and RBD [20]. CpG has also been included in LDH-based formulations to enhance the adjuvanticity of the nanosystem [21,22]. Noteworthy among these efforts are the studies by Zhang et al. [23] and Chen et al. [24], who loaded LDHs with three distinct antigens. Across all these studies, the LDHs used as an adjuvant produced a milder inflammatory response at the injection site compared to that of Alum, while achieving effective humoral and cellular immune responses [25,26,27,28,29,30].

It is important to note that until now, all the reported LDH-based nanovaccines have been developed through passive conjugation, thus relying on electrostatic, hydrophobic, and/or van der Waals interactions. Moreover, most of the reports have used protein molecules as antigens. Active conjugation, in contrast, is a process where a covalent bond is formed between two molecules, creating a stronger interaction [31]. Intended as a proof of concept for gene delivery systems, the grafting of a 12-nucleotide-long, single-stranded sequence on an LDH using a carbodiimide crosslinker is an example of a potential therapeutic agent produced by covalent bonding [32]. This type of approach has not been pursued with LDHs to develop a subunit nanovaccine based on peptide molecules.

Therefore, this work focused on creating a candidate peptide-based nanovaccine against SARS-CoV-2 covalently bound onto LDH nanoparticles. The proposed formulation employed active conjugation mediated by reductive amination chemistry. To the best of our knowledge, this is the first report on creating nanovaccines using the covalent bonding of an antigen to the LDH surface, providing relevant data to further develop nanovaccines through active conjugation.

## 2. Materials and Methods

All the following chemical reagents used were of analytical grade: magnesium nitrate hexahydrate (98%, 256.4 g/mol, Sigma-Aldrich, St. Louis, MO, USA), aluminum nitrate nonahydrate (98%, 375.1 g/mol, Sigma-Aldrich, St. Louis, MO, USA), APTES ((3-aminopropyl)triethoxysilane) (98%, 221.4 g/mol, Sigma-Aldrich, St. Louis, MO, USA), GPS (3-glycidoxypropyltrimethoxysilane) (98%, 236.3 g/mol, Sigma-Aldrich, St. Louis, MO, USA), CPS ((3-chloropropyl)trimethoxysilane) (97%, 198.7 g/mol), glutaraldehyde (25%, 100.1 g/mol, Sigma-Aldrich, St. Louis, MO, USA), L-lysine monohydrate (98.5%, 164.2 g/mol, St. Louis, MO, USA), glycine (99%, 75.1 g/mol, St. Louis, MO, USA), ninhydrin (178.1 g/mol, St. Louis, MO, USA), hydrindantin (97%, 322.3 g/mol, St. Louis, MO, USA), sodium triacetoxyborohydride (97%, 211.9 g/mol, St. Louis, MO, USA), sodium acetate trihydrate (99%, 136.1 g/mol, St. Louis, MO, USA), MOPS (3-(N-morpholino)propanesulfonic acid) (99.5%, 209.3 g/mol, St. Louis, MO, USA), trifluoroacetic acid (99%, 114.0 g/mol, St. Louis, MO, USA), sodium hydroxide (97%, 40.0 g/mol, PQF, Nuevo Leon, Mexico), sodium chloride (99%, 58.4 g/mol, PQF, Nuevo Leon, Mexico), ethanol (99.9%, 46.1 g/mol, PQF, Nuevo Leon, Mexico), dimethyl sulfoxide (99.9%, 78.1 g/mol, PQF, Nuevo Leon, Mexico), acetonitrile (99.9%, 41.1 g/mol, PQF, Nuevo Leon, Mexico), hydrochloric acid (35.5%, 36.5 g/mol, Fermont, Nuevo Leon, Mexico), glacial acetic acid (99.7%, 60.1 g/mol, Fermont, Nuevo Leon, Mexico), and sodium carbonate (99.5%, 106.0 g/mol, Jalmek, Nuevo Leon, Mexico). A SpectraPor 4 dialysis membrane (12–14 kDa, MWCO) was purchased from Repligen (Rancho Dominguez, CA, USA). Two peptides identified as P_2_ (HADQLTPTWRVYSTGSNV) and P_5_ (DSFKEELDKYFKNHTS) were obtained from Synpeptide (Shanghai, China). Both peptides have been described as candidates against SARS-CoV-2 [33].

### 2.1. Synthesis of Magnesium-Aluminum LDH (Mg-Al LDH) Nanoparticles (NPs)

LDH NPs were synthesized with a Mg/Al 3:1 molar ratio using a combined coprecipitation and hydrothermal method based on the procedure described by Xu et al. [34]. The coprecipitation was performed by rapidly adding 10 mL of solution containing 0.15 M Mg(NO_3_)_2_·6H_2_O and 0.05 M Al(NO_3_)_3_·9H_2_O to 15 mL of a solution containing 0.4 M NaOH (already under stirring with a vortex mixer) and mixing the resulting suspension for 10 min more. This precipitate was washed twice using centrifugation–resuspension cycles at 21,200× *g* for 5 min, replacing the supernatant with deionized water, and resuspending the sample for 5 s with an ultrasonic probe at 20% amplitude. For the hydrothermal process, the suspension (25 mL) was transferred to a PTFE-lined stainless steel reactor and heated at 100 °C for 2 h in an oil bath or in an oven. Afterward, the LDH suspension was washed again by centrifuging at 21,200× *g* for 30 min and resuspended in ethanol using ultrasonication. To determine the concentration of LDHs synthesized, 1 mL of suspension was centrifuged, the supernatant discarded, and the sediment weighed after being dried in a desiccator for 12 h.

### 2.2. Functionalization of LDHs with Organosilanes

Based on the concentration of LDHs and their average size, the available surface area (A_S_) per nanoparticle for functionalization was calculated. In parallel, the circular area (A_C_) occupied by each functional molecule was estimated using its molecular weight and density. By determining the number of A_C_ units required to uniformly cover the A_S_, the concentration of functional molecules (e.g., APTES) needed to form a monolayer coating on the LDH NPs was estimated. The methodology to functionalize LDHs followed the protocols reported by Hermanson [31] and Chhetri et al. [35]. A 3 mL solution containing 95% ethanol and 5% deionized water was prepared, into which 1% APTES (75 µL, equivalent to 100 monolayers) was dissolved and hydrolyzed for 5 min. This solution was then combined with 3 mL of LDH stock suspension (3 mg/mL) on a stirring plate set to 700 rpm. Five methods were explored by varying the contact time (CT, between 0 and 18 h) at room temperature and reflux time (RT, 75 °C, between 0 and 2 h), labeled as A (CT = 0 h, RT = 2 h), B (CT = 1 h, RT = 2 h), C (CT = 1 h, RT = 0 h), D (CT = 18 h, RT = 2 h), and E (CT = 18 h, RT = 0 h). Afterward, the samples were transferred to vials, and the nanoparticles were washed with ethanol via centrifugation at 21,200× *g* for 15 min, followed by dispersion in an ultrasonic bath. This washing process was repeated twice. Finally, a stock solution with a concentration of 3 mg/mL in ethanol was prepared. In addition to the functionalization with APTES, alternative approaches using GPS and CPS were tested to reduce the number of steps before active conjugation. These nanoparticles were aminated with lysine with a concentration equivalent to 100 monolayers in 20 mM carbonate buffer at pH 10, with constant stirring for 24 h. Finally, the concentration of primary amines on the surface of the nanoparticles was determined using the ninhydrin assay described below.

### 2.3. Reductive Amination with Glutaraldehyde

The aminated LDH nanoparticles (LDH-NH_2_) at a concentration of 1 mg/mL were reacted with 1% glutaraldehyde (GTA) in the presence of the reducing agent sodium triacetoxyborohydride (STAB, 1%). The reaction was performed in 20 mM carbonate buffer at pH 10 for 0.5, 1.0, 2.0, and 4.0 h to optimize the reductive amination time. Afterward, the suspensions were dialyzed using a 12–14 kDa MWCO membrane. Dialysis was conducted in three steps: an initial 5 h step with 45 mL of the respective buffer, followed by two additional steps lasting 18 and 5 h, each with fresh buffer replacement. The resulting aldehyde-functionalized nanoparticles (LDH-CHO) were recovered from the microdialysis tube, and the residual primary amine content was quantified using the ninhydrin assay. All reaction and dialysis solutions were degassed for 5 min prior to use.

### 2.4. Active Conjugation of Lysine and Peptides

The LDH-CHO nanoparticles (0.5 mg/mL) were dispersed in one of three buffers: 20 mM MOPS at pH 7.2, 20 mM carbonate at pH 10.0, or 20 mM phosphate at pH 7.0. Each buffer contained lysine (Lys) at 5× molar excess (relative to the original concentration of amino groups) and an excess of STAB (5×). The reaction mixtures were stirred on a rotary mixer at 30 rpm for 18 h. The resulting conjugates were dialyzed in microtubes using a 12–14 kDa MWCO membrane against 45 mL of dialysis buffer containing 500 mM NaCl to disrupt potential electrostatic interactions between the LDHs and Lys. Two additional dialysis steps lasting 18 and 5 h, respectively, were performed, replacing the NaCl buffer with deionized water. After dialysis, the lysine-functionalized LDH nanoparticles (LDH-Lys) were recovered, and the primary amine content was quantified using the ninhydrin assay. All reaction and dialysis solutions were degassed for 5 min prior to use.

**Figure 1:**
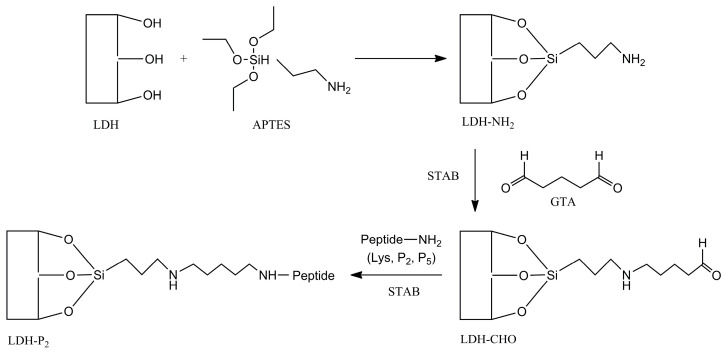
Reaction scheme to covalently bind peptides to surface of aminated LDH using APTES. Glutaraldehyde (GTA) was used as crosslinker, and STAB (sodium triacetoxyborohydride) as reducing agent.

Once the best buffer was selected to perform the active conjugation process, the same protocol as described previously was used to bind the peptides P_2_ and P_5_ onto LDH-CHO (Figure 1), with the final redispersion of the candidates in 10 mM MOPS (pH 7.0) containing 150 mM NaCl. After recovering the LDH-P_2_ and LDH-P_5_ conjugates, a portion of each sample was analyzed to determine the concentration of peptides bound on LDH-CHO using HPLC. For this analysis, the particles were fully dissolved in 1 M HCl for 15 min. After neutralization with NaOH, the released peptides were quantified using reverse-phase chromatography (details provided below).

### 2.5. Quantification of Primary Amines and Peptides

The concentration of amino groups on the surface of the LDHs was analyzed using the ninhydrin method reported by Moore & Stein [36]. A calibration line was prepared with concentrations ranging from 0 to 0.2 mM using glycine as the standard. For analysis, 20 mg of ninhydrin and 3 mg of hydrindantin were dissolved in 0.75 mL of DMSO, and then 0.25 mL of acetate buffer (4 M, pH 5.5) was added. For quantification, a mixture was prepared containing 0.02 mL of the LDH sample (LDH-NH_2_, LDH-CHO, or LDH-Lys) or a standard, 0.08 mL of deionized water, and 0.05 mL of ninhydrin solution. The mixture was then heated for 15 min in a dry bath at 90 °C. After this, it was allowed to cool for 5 min, diluted with 0.15 mL of deionized water, and the absorbance of each sample was measured using a UV-Vis spectrophotometer (Genesys 50, Thermo Scientific, Waltham, MA, USA).

The peptide concentration was quantified using a liquid chromatograph (1260 Infinity, Agilent Technologies, Santa Clara, CA, USA) equipped with a diode array detector (DAD). A stock of peptides at different concentrations (P_2_ or P_5_, from 25 to 100 µg/mL) was used to generate a calibration line to quantify their concentrations once released during the solubilization of the nanoconjugates (LDH-P_2_ or LDH-P_5_). A total of 100 µL of sample was injected into an XDB-C18 column (150 mm × 4.6 mm) previously equilibrated with buffer A (0.1% trifluoroacetic acid in water). The flow rate was 0.5 mL/min, detection was set to 214 nm, and a gradient was applied from 100% to 0% buffer A in 10 min. Buffer B (0.1% trifluoroacetic acid in acetonitrile) was used for this purpose.

### 2.6. Immunization Scheme in Mice

To assess immunogenicity, 12-week-old female BALB/c mice (acquired from Inotiv, West Lafayette, IN, USA) received subcutaneous injections of 200 µL containing either LDH-P_2_, LDH-P_5_, Al(OH)_3_-P_2_, or Al(OH)_3_-P_5_, each with 5 µg of antigen. Al(OH)_3_-P_2_ and Al(OH)_3_-P_5_ formulations were prepared by incubating the respective peptide (5 µg) with alum adjuvant diluted at a 1:5 ratio (G Biosciences (St. Louis, MO, USA), cat no. 786-1215). The injections were administered on days 1 and 15. Peripheral blood samples were collected via tail vein puncture on days 0, 14, and 29. The collected blood samples were centrifuged at 1200× *g* for 10 min, and the sera were separated and stored at −20 °C for further analysis. The Ethics Institutional Committee approved this protocol under the code CEID-2020-07R1.

### 2.7. IgG Titer Quantification by ELISA

Anti-P_2_ and anti-P_5_ IgG titers induced by immunization with P_2_ or P_5_ in the alum adjuvant or bound to the LDHs (LDH-P_2_ or LDH-P_5_) were determined by ELISA using serial dilutions of the test sera following the booster dose. Polystyrene 96-well plates were coated overnight with 100 ng of P_2_ or P_5_ per well diluted in 0.2 M carbonate buffer. The plates were blocked overnight at 4 °C with a 5% fat-free milk solution. After blocking, serial dilutions of the sera were applied to the wells and incubated overnight at 4 °C. After each incubation step, the plates were washed three times with PBST (1× PBS + 0.5% Tween-20). For secondary labeling, horseradish peroxidase-conjugated anti-mouse IgG (Sigma-Aldrich, St. Louis, MO, USA) was applied, followed by 2 h incubation at room temperature. The reaction was developed by adding a substrate solution consisting of 0.3 mg/L ABTS and 0.1 M H_2_O_2_, and the plates were incubated at room temperature for 30 min. Absorbance was measured at 405 nm using a Multiskan Ascent microplate reader (Thermo Fisher Scientific, Waltham, MA, USA).

### 2.8. Cytotoxicity Assay

The cytotoxicity of the synthesized LDH-P_2_ nanovaccine was evaluated using the HEK-293T (ATCC (Manassas, VA, USA), cat. no. CRL-3216; https://www.atcc.org/products/crl-3216 (accessed on 1 May 2025)) cell line and the resazurin assay. A total of 2 × 10^5^ cells was seeded in triplicate in a 96-well plate containing DMEM medium supplemented with 10% fetal bovine serum and 1% penicillin/streptomycin. The cells were incubated at 37 °C in a 5% CO_2_ atmosphere. After 24 h, the cells were washed once with 1× PBS and treated with LDH, LDH-NH_2_, LDH-CHO, and LDH-P_2_ at concentrations ranging from 10 to 500 µg/mL. A positive control was implemented with 40 mM H_2_O_2_, and a negative control experiment was performed using the vehicle alone. After 48 and 72 h of incubation, the cells were washed again and exposed to 30 µg/mL of resazurin for 4 h. Fluorescence (excitation at 560 nm, emission at 590 nm) was measured using a FlexStation II microplate reader (Molecular Devices, San Jose, CA, USA).

### 2.9. Characterization

The hydrodynamic particle size (d_z_), the polydispersity index (PdI), and the ζ potential were characterized by measuring dynamic (DLS) and electrophoretic light scattering (ELS) using the particle analyzer ZetaSizer Pro (Malvern Panalytical, Malvern, UK). Morphology was characterized with a transmission electron microscope (TEM) JEM-2001 (JEOL, Akishima, Japan). The structure of the materials was studied using a powder X-ray diffractometer (XRD) D2 phaser (Bruker, Billerica, MA, USA) under the following operating conditions, a scanning range from 2° to 70° (2Θ), a scanning speed of 1.8°/min, a voltage of 36 kV, and a filament current of 35 mA, with Cu Kα radiation at λ = 0.154184 nm. Thermogravimetric analysis (TGA) was performed using the analyzer TGA-550 (TA Instruments, New Castle, DE, USA), with a heating rate of 10 °C/min in a range from 25 to 1000 °C. Fourier transform infrared (FTIR) spectroscopy under attenuated total reflectance (ATR) with a diamond crystal and covering a range from 500 to 4000 cm^−1^ was recorded with an FTIR spectrometer Nicolet 6700 having a DTGS detector (Thermo Scientific, Waltham, MA, USA), with a resolution of 4 cm^−1^ and 16 scans per sample. A calorimetric study was performed using a differential scanning calorimeter (DSC) (8500, PerkinElmer, Waltham, MA, USA) over a temperature range of 25–400 °C, with a heating rate of 10 °C/min and a single heating cycle. Elemental composition analysis was performed using a dual-beam focused ion beam/scanning electron microscope (FIB/SEM) (Helios Nanolab 600, Thermo Scientific, Waltham, MA, USA) operated at an accelerating voltage of 20 kV using a gallium ion source for the ion beam.

### 2.10. Statistical Analysis

All the experiments were performed in triplicate, and the data are presented as mean ± SD. Statistical analysis was conducted using one-way ANOVA in Microsoft Excel^®^ (Microsoft 365, Microsoft Corporation, Redmond, WA, USA), with *p* = 0.05 to establish significance.

## 3. Results and Discussion

### 3.1. LDH Nanoparticles Synthesized

**Figure 2:**
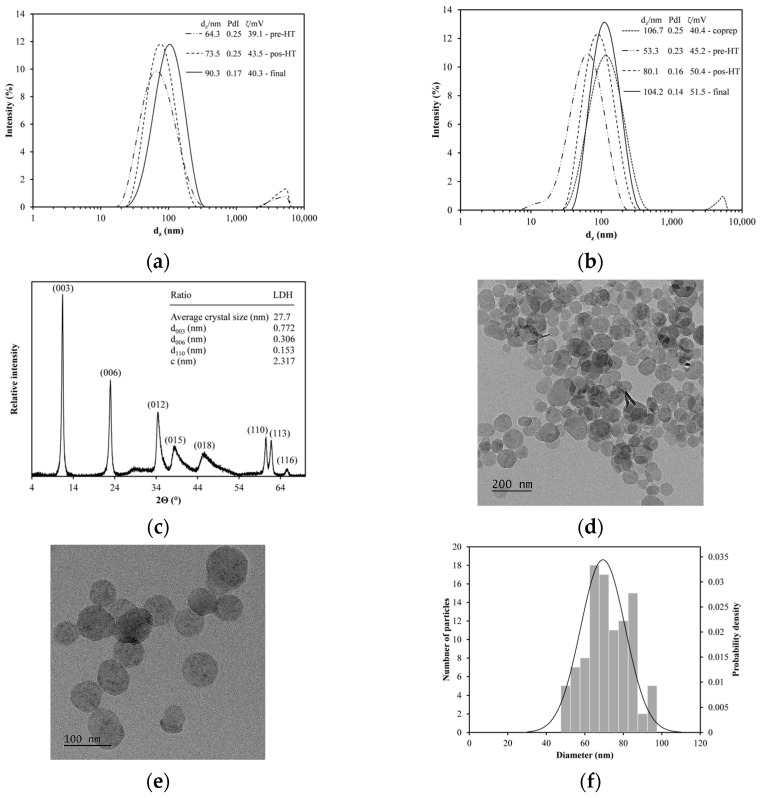
Synthesis of Mg/Al layered double hydroxides (LDH). (**a**,**b**) size distribution of LDHs synthesized in mineral oil bath and in oven, respectively; (**c**) XRD diffraction patterns; (**d**,**e**) TEM images of a typical final suspension, with scale bars of 200 nm and 100 nm, respectively; and (**f**) size distribution of LDHs after analyzing 100 particles.

The LDHs were produced using a sequential synthesis method consisting of coprecipitation and hydrothermal treatment (HT) with a Mg^2+^/Al^3+^ molar ratio of 3:1. Figure 2 organizes their characterization through various techniques. Figure 2a,b shows the particle size distribution at different stages during the synthesis: the particles obtained after coprecipitation (coprep), the particles washed before hydrothermal treatment (pre-HT), the particles after hydrothermal treatment (post-HT), and the particles washed after completing all the previous stages (final).

The hydrothermal treatment was conducted in an oil bath (Figure 2a) or an oven (Figure 2b). The d_z_ and PdI values were similar, with only slight differences observed in the ζ potential. While both the heating methods allow for producing LDHs with comparable sizes, the uniform temperature in the oven rendered LDHs with narrower size distributions. The particles chosen for subsequent studies were those produced in an oven, with values of d_z_, PdI, and ζ potential measuring 104.2 ± 6 nm, 0.14 ± 0.03, and 51.5 ± 6 mV, respectively. These results indicate monodisperse and stable particles, achieving LDH yields of 5 mg/mL in aqueous suspensions. The d_z_ and ζ potential of the synthesized LDHs are consistent with those reported by Xu et al. [34], who also obtained monodisperse nanoparticles. Additionally, the yield is in accordance with values reported in other studies [37,38].

Figure 2c shows the XRD pattern of the LDHs, confirming that the synthesized nanoparticles exhibit the hydrotalcite crystalline phase. Phase identification was performed using the PDF-2 database (ICDD, PDF #35-0965) via JADE 6 software (MDI, Newton Square, PA, USA). This fact is evidenced by the characteristic crystallographic planes of the material, supported by a table reporting the parameters of the hexagonal unit cell. The sharp and well-defined peaks further validate the crystalline quality of the synthesized LDHs.

Figure 2d,e shows TEM images with scales of 200 and 100 nm, respectively. Additionally, the histogram presents the nanoparticle size distribution (Figure 2f), with an average size of 69.4 ± 11.6 nm. A Kolmogorov–Smirnov (K-S) test [39] was conducted to determine whether the size distribution of the synthesized LDH nanoparticles followed a normal distribution. The maximum difference between the observed and expected cumulative distributions was 0.072, which is lower than the critical value of 0.136 for a sample size of 100 at a 95% confidence level. Therefore, the synthesized LDHs are uniform in size, highlighting effective control during the synthesis process.

### 3.2. LDHs Functionalized by Silanization

Five different silanization methods and three organosilanes were tested for the functionalization of the LDHs. The primary distinction between these compounds is their functional groups; APTES contains an amino group, GPS has an epoxide group, and CPS features an alkyl halide group. The last two were subsequently aminated with Lys in 20 mM carbonate buffer. APTES has been commonly used as a silanization agent for LDHs, either with surfactants [40,41] or without them [42,43,44], but not in the development of nanovaccines. GPS attached to triazabicyclodecene has been used to silanize surfactant-modified LDHs, but exclusively for catalytic applications [45]. In contrast, CPS has not been reported for the silanization of LDHs in any application.

**Figure 3:**
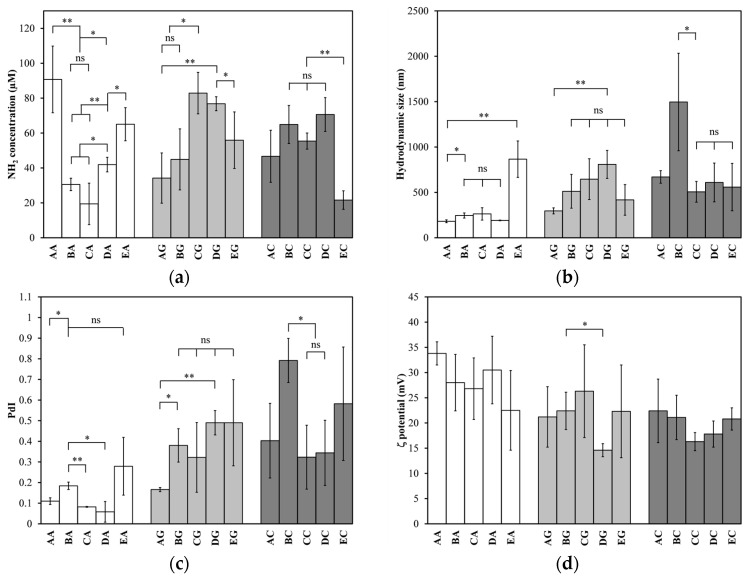
Silanization of LDHs. (**a**) Primary amine concentration (µM); (**b**) hydrodynamic size (nm); (**c**) polydispersity index (PdI); and (**d**) ζ potential (mV) as a function of five methods tested with three organosilanes. X-axis labels: first letter is one of five methods (from A to E), while second is organosilane (A is APTES, G is GPS, and C is CPS), e.g., AA is method A (2 h reflux) with APTES. Significant differences indicated as * *p* < 0.05, ** *p* < 0.01, and ns (not significant).

The quantification of primary amines in the functionalized LDHs was performed within 24 h. The results of each functionalization method and organosilane tested in triplicate are shown in Figure 3a. APTES generated the highest concentration of primary amines, 91 ± 19 µM, with method A (2 h reflux). For GPS, the best outcome was achieved with method C (1 h reaction), yielding a concentration of 83 ± 12 µM. For CPS, method D (18 h reaction, then 2 h reflux) was optimal, resulting in a concentration of 71 ± 10 µM.

Figure 3b shows the hydrodynamic sizes of the aminated LDHs, while Figure 3c shows their polydispersity indices (PdIs). For APTES, the particle size ranged from 180 to 866 nm, having a PdI below 0.3 with method E (18 h reaction) and lower than 0.2 with the other four methods. For GPS, the size ranged from 296 to 650 nm, with a PdI below 0.5. Lastly, for CPS, the particle size varied from 506 to 1496 nm, with a PdI of up to 0.8. These results indicate that the absence of a charged functional group on the silane leads to an increase in both the hydrodynamic size and its PdI, with visible aggregates observed in suspensions of CPS-functionalized LDHs. In contrast, the LDHs silanized with APTES exhibited the lowest PdI, indicating more uniform functionalization.

Figure 3d shows the ζ potential of the aminated LDHs with the three silanizing agents tested. Their values ranged from 14 to 34 mV, lower than those of the unmodified LDHs (51.5 ± 6.0 mV). These lowered values indicate that during functionalization, as the silanes bound to the LDHs forming covalent bonds with the hydroxyl groups available, they decreased the density of the positively charged groups on the surface, and consequently lowered the ζ potential. This decrease in ζ potential negatively affected the colloidal stability of the system, increasing the probability of forming aggregates, which is in line with the results obtained for hydrodynamic size and PdI. The aminated LDHs were in suspension at pH seven. As reference, the point of zero charge for several LDHs has been reported in a pH range from eight to twelve [46].

It is worth noting that no studies have been reported that follow changes in d_z_, PdI, and ζ potential during the silanization of LDHs. Moreover, the use of the ninhydrin reaction to quantify the concentration of primary amines on the surface of LDHs has not been documented.

These findings were crucial in selecting the most suitable organosilane and functionalization method. Since modification is intended for use in the formulation of a nanovaccine, APTES was chosen based on the results, as it rendered amino-functionalized LDHs (LDH-NH_2_) with a reliable hydrodynamic size and a low PdI, more uniform functionalization, and better colloidal stability. Moreover, when comparing APTES with GPS and CPS using method A, the former produced the highest amino group concentration, the smallest hydrodynamic size and PdI, and the highest ζ potential. Therefore, this method of functionalization with APTES was selected for further studies.

**Figure 4:**
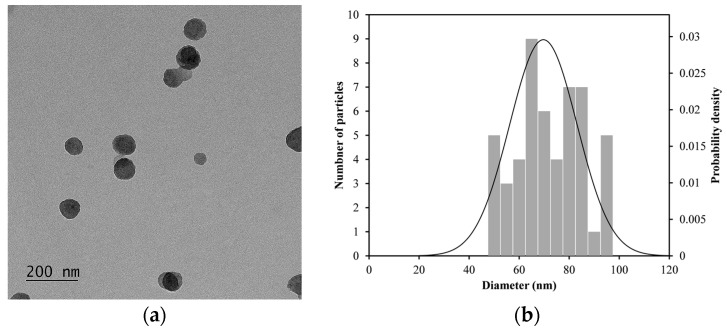
(**a**) TEM image of LDH-NH_2_ after functionalizing LDH with APTES using method A. (**b**) Size distribution of LDH-NH_2_, measured after analyzing 50 particles.

Figure 4a shows a TEM image of LDH-NH_2_ with a 100 nm scale, while Figure 4b shows a corresponding histogram displaying their size distribution. The average particle size of LDH-NH_2_ was 69.7 ± 13.5 nm, while the hydrodynamic size was 181.4 ± 15.8 nm with a PdI of 0.16 ± 0.09. This difference is expected due to the larger molecular size of the aminopropyl groups (after the condensation of APTES) now attached to the LDHs compared to those of the original hydroxyl groups. These moieties can interact with more distant water molecules in the aqueous dispersant, while the PdI value is representative of homogeneous particle distribution.

**Figure 5:**
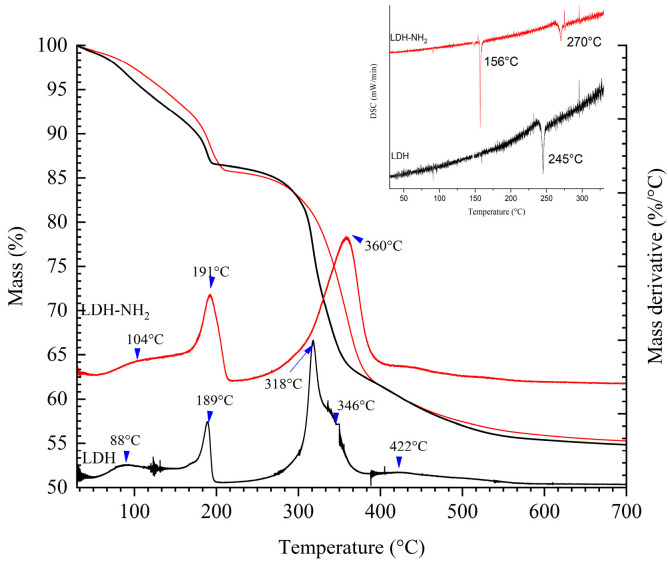
Thermogravimetric analysis showing loss percentage curves and mass loss rate of LDHs and LDH-NH_2_ (LDHs functionalized with APTES using method A).

Figure 5 shows thermogravimetric analysis (TGA) curves of LDHs, displaying mass loss percentage curves and the mass loss rate. Five significant mass loss events occurred at 88, 189, 318, 346, and 422 °C with mass loss percentages of 5, 13, 17, 8, and 5%, respectively, characteristic of this material. These losses correspond to the removal of adsorbed water, the loss of interlayer water and initial dehydroxylation, complete dehydroxylation, the decarbonation of intercalated anions, and the formation of mixed oxides [47]. Figure 5 also shows the thermogravimetric analysis curves of LDH-NH_2_, displaying three distinct mass loss events at 102, 191, and 360 °C, with corresponding mass loss percentages of 3, 9, and 23%, respectively. The functionalization of the LDHs with APTES caused a shift in the temperatures at which these changes occur, indicating increased thermal stability. Silane groups react with hydroxylated surfaces, reducing water adsorption and stabilizing the material. In contrast, the DSC curve recorded between 25 °C and 400 °C displays a single thermal event at 245 °C for the LDHs, corresponding to the dihydroxylation process. The discrepancy between TGA and DSC in terms of the number of thermal events and their respective temperatures suggests that the surface and interlayer water molecules are physiosorbed. Their evaporation or release requires smaller energy inputs, which are not detected by DSC. The LDH-NH_2_ sample exhibits two prominent peaks at 156 and 270 °C. The first peak is attributed to the decomposition of the organic phase, associated with APTES adsorbed on the LDH surface, while the second peak likely corresponds to a delayed dihydroxylation event caused by the presence of APTES within the LDH structure. This temperature shift provides compelling evidence for the formation of covalent bonds due to the silanization process.

Using the TGA data, the concentration of APTES bound to the LDHs was calculated as follows. Per each initial mg of sample, 0.53 mg of LDH and 0.67 mg of LDH-NH_2_ remained after TGA. Both the samples exhibited similar losses (e.g., dehydroxylation and decarbonation); therefore, the mass difference was attributed to silanization, corresponding to 0.632 µmol of APTES per mg of LDH. Alternatively, considering the decomposition of LDH-NH_2_ between 193 and 359 °C, the mass loss from 12 to 31% yields an estimate of 0.858 µmol of APTES per mg of LDH. However, while this second approach accounts for the degradation of organic residues, the percentage difference inherently includes mass loss due to dehydroxylation, which leads to the overestimation of the amount of APTES bound to the LDHs.

To corroborate the formation of covalent bonds between APTES and the LDHs, experiments were carried out following the methodology described in Section 2.2 using the conditions of method A, but at 25 °C. Under these conditions, without thermal activation, weak interactions such as electrostatic forces or hydrogen bonding between APTES and the LDH surface may occur, leading to physisorption. The material obtained through this process was designated as LDH-NH_2_^P^.

**Figure 6:**
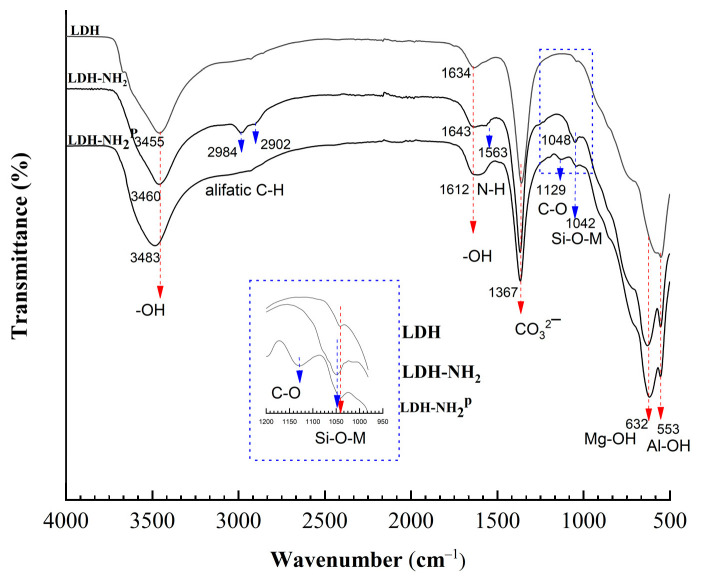
FTIR spectra of LDH, LDH-NH_2_ (method A), and LDH-NH_2_^P^ (method A at 25 °C).

Figure 6 shows the FTIR spectra of the LDH, LDH-NH_2_, and LDH-NH_2_^P^ samples. The LDH spectrum displays characteristic bands corresponding to –OH stretching (3668 and 3455 cm^−1^), interlayer water bending (1634 cm^−1^), CO_3_^2−^ vibrations (1367 cm^−1^), Al–OH/Mg–OH bond vibrations (1040 cm^−1^), CO_3_^2−^ torsion (709 cm^−1^), and Mg–O/Al–O translational vibrations (594 and 553 cm^−1^) [48]. The FTIR spectrum of LDH-NH_2_ confirms the incorporation of APTES, as evidenced by the C–H stretching of alkyl groups (2902–2984 cm^−1^), the N–H bending of amine groups (1563 cm^−1^), and Si–O–M stretching (1048 cm^−1^) [35]. In the LDH-NH_2_^P^ sample, bands corresponding to the Si–O–M stretching (1042 cm^−1^) and C–H stretching of the alkyl chain are present, but show reduced intensity. Additionally, a C–O band appears at 1129 cm^−1^, which may be attributed to unreacted groups. The presence of well-defined Si–O–M, C–H, and N–H bands, along with the decreased intensity of –OH bands in LDH-NH_2_ due to their reaction with APTES (a change not observed in LDH-NH_2_^P^), supports the conclusion that covalent bonds are formed between the surface –OH groups of the LDHs and APTES in the LDH-NH_2_ sample.

### 3.3. Binding Glutaraldehyde to LDH-NH_2_

**Figure 7:**
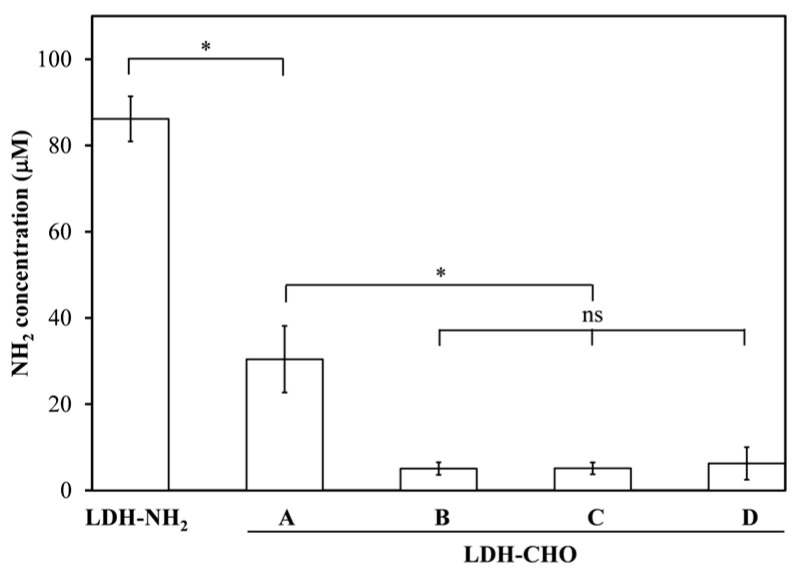
Binding GTA onto LDH-NH_2_ surface, measured in terms of amino groups concentration, to generate LDH-CHO. Reaction times of 0.5, 1.0, 2.0, and 4.0 h correspond to axis labels A, B, C, and D, respectively. Significant differences indicated as * *p* < 0.05, while ns denotes no significant difference.

After selecting APTES as the best silanizing agent, the next step was to use a linker that enables the binding of an amino-containing molecule onto the LDH-NH_2_ surface. For this purpose, glutaraldehyde (GTA), a dialdehyde, was selected to run a reductive amination reaction to obtain aldehyde-functionalized LDHs (LDH-CHO). This study to determine the optimal reaction time was run for 0.5, 1.0, 2.0, and 4.0 h. The results (Figure 7) indicated that 1 h was sufficient to decrease the amino group concentration by more than 90%.

### 3.4. Binding Lysine or Peptides to LDH-CHO

**Figure 8:**
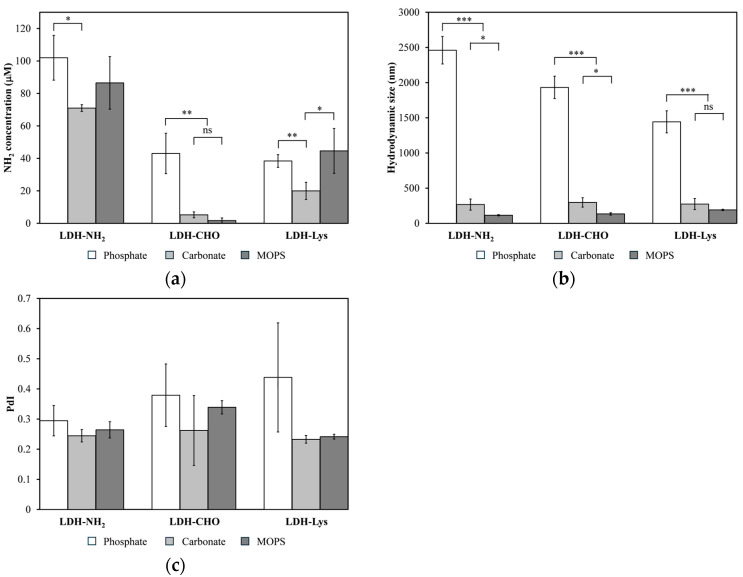
Effects of phosphate, carbonate, and MOPS buffers on (**a**) concentration of primary amines; (**b**) hydrodynamic size (d_z_); and (**c**) polydispersity index (PdI) of LDH-NH_2_, along with their influence on reactions to produce LDH-CHO and LDH-Lys. Significant differences indicated as * *p* < 0.05, ** *p* < 0.01, *** *p* < 0.001, and ns (not significant).

Lysine (Lys) was first bound onto LDH-NH_2_ using GTA as a crosslinker under a reductive amination scheme. The reaction was conducted under different buffer conditions: 20 mM MOPS at pH 7.2, 20 mM carbonate at pH 10.0, and 20 mM phosphate at pH 7.0. Figure 8 shows the results for each reaction step, analyzing the concentration of primary amines, the hydrodynamic size, and PdI. Irrespective of the buffer used, the amine concentration decreased significantly when GTA reacted with LDH-NH_2_ to form LDH-CHO, indicating a successful reaction. When adding Lys to react with LDH-CHO, the amine concentration increased significantly with the carbonate and MOPS buffers. On the other hand, phosphate buffer produced the largest hydrodynamic sizes for all the particles (LDH-NH_2_, LDH-CHO, and LDH-Lys). In contrast, the MOPS buffer maintained the LDH-Lys size below 200 nm with a PdI of 0.24 ± 0.01.

Previous reports have shown that phosphate ions strongly adsorb onto the LDH surface, shifting the ζ potential toward negative values at phosphate concentrations above 0.8 mM [49] or at a pH above six [50]. This shift was confirmed in this study using 20 mM phosphate; for instance, in LDH-NH_2_, the ζ potential shifted from 33.9 ± 2.3 to −14.9 ± 1.0 mV. This change in ζ potential promoted the aggregation of LDH-NH_2_, which persisted throughout the reaction steps. The phosphate ions adsorbed on the LDHs decreased the surface area, and thus contributed to the poor incorporation of Lys molecules onto LDH-CHO.

Therefore, MOPS was selected as the reaction buffer for binding peptide molecules to LDH-NH_2_. The results of peptide concentration (HPLC), hydrodynamic size, and PdI of the resulting LDH-P_2_ and LDH-P_5_ conjugates are summarized in Table 1.

**Figure 9:**
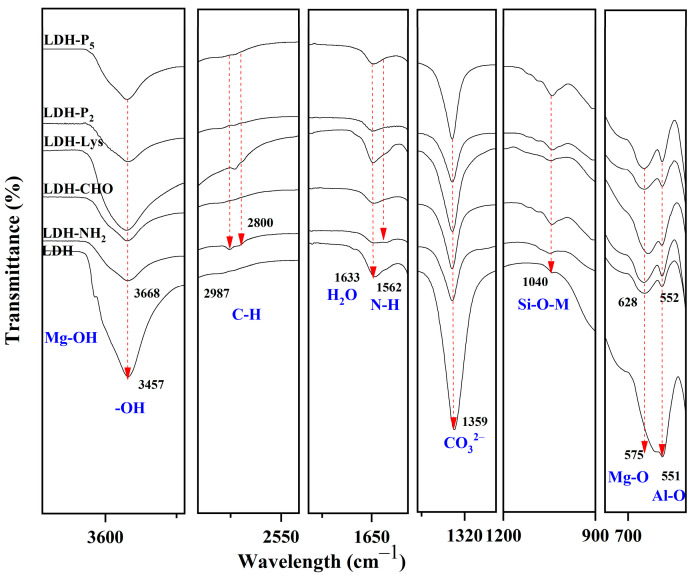
FTIR spectra of LDHs silanized with APTES (LDH-NH_2_), activated with GTA (LDH-CHO), and conjugated with either lysine (LDH-Lys) or peptides P_2_ (LDH-P_2_) and P_5_ (LDH-P_5_).

To confirm the conjugation of LDH particles at each modification stage, FTIR analysis was performed on dry samples (Figure 9). In the FTIR spectrum of LDH-CHO, the presence of bands at 1638 cm^−1^ (C=N), 1564 cm^−1^ (N–H), and 1041 cm^−1^ (Si–O) suggests partial Schiff base formation (–C=N–), characteristic of amine–aldehyde reactions. Additionally, the C–N bond vibration (906 cm^−1^) confirms functionalization with GTA [51]. The FTIR spectrum of LDH-Lys shows alkyl groups signals from APTES and lysine (2938 and 2800 cm^−1^) and Schiff base formation (–C=N–) at 1640 cm^−1^ [51]. The FTIR spectrum of LDH-P_2_ presents bands at 1538 cm^−1^ (N–H, Amide II) and 1083 cm^−1^ (C–O, ether), while LDH-P_5_ shows a band at 1110 cm^−1^, both confirming peptide bond formation through the amide I and II bands [33]. Throughout each modification stage, the FTIR spectra exhibit shifts and reductions in characteristic LDH bands, indicating the formation of covalent bonds on its surface. These findings confirm the success of the proposed conjugation route.

**Figure 10:**
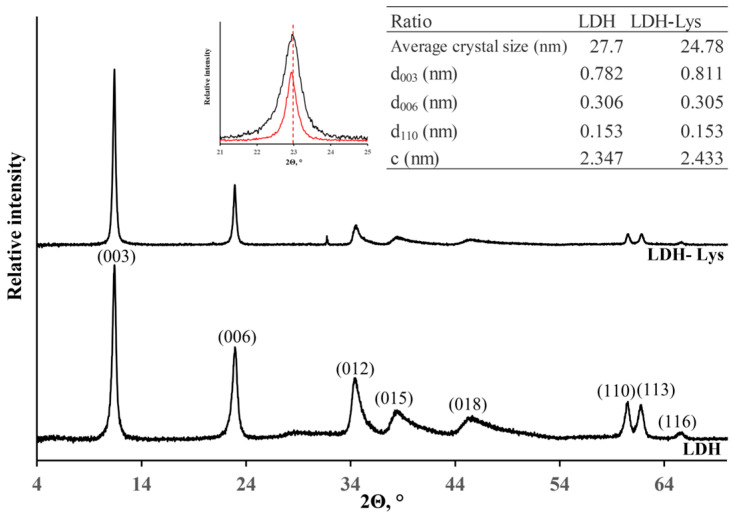
XRD diffraction patterns of LDHs conjugated with lysine (LDH-Lys). In the inset, the red line represents LDH, while the black line is LDH-Lys.

Figure 10 presents the XRD patterns of LDH and LDH-Lys samples. In the LDH-Lys sample, a noticeable shift of the (003) and (006) reflections toward lower 2Θ values is observed relative to the raw LDHs, indicating the expansion of interlayer spacing. This shift suggests the successful intercalation of organic molecules between the LDH layers. Specifically, the interlayer distance for the (003) plane increased from 0.782 to 0.811 nm. In contrast, no significant change was observed in the (110) plane, indicating that the Mg/Al ratio in the brucite-like layers was preserved. Overall, the LDH-Lys diffractogram reveals reduced peak intensity and broader reflections, consistent with decreased crystallinity due to the structural modifications induced by organic conjugation [52].

The results presented in Table 2 confirm the presence of elements such as C, indicative of the organic phases, as well as Mg, Al, and Si, which are associated with the inorganic LDH framework, at each stage of the reaction. In addition, as shown in Table 3, a progressive decrease in the Mg/Al ratio is observed, attributed to the incorporation of organic compounds, which increase their relative proportion within the sample. In contrast, the C/LDH ratio rises across the sample series. These findings support the structural integrity of the LDHs throughout the nanovaccine synthesis process and confirm the successful incorporation of organic species into the final material.

### 3.5. Cytotoxicity and Immunogenicity

**Figure 11:**
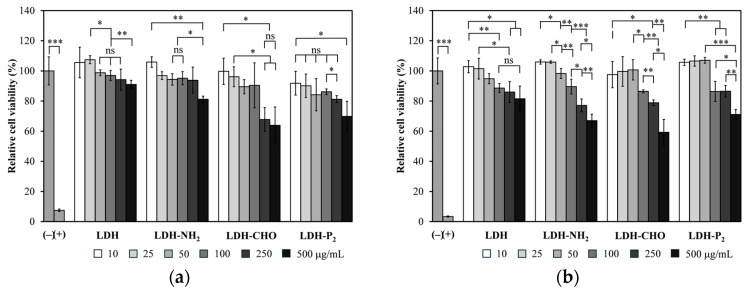
Toxicity assessment of LDHs and the modifications. Viability of HEK-293T cells treated for (**a**) 48 h and (**b**) 72 h was analyzed using resazurin reduction assay, which measures conversion to resorufin by metabolically active cells. Cells treated with vehicle served as negative toxicity control (–), while H_2_O_2_ was used as positive control (+). Significant differences indicated as * *p* < 0.05, ** *p* < 0.01, *** *p* < 0.001, and ns (not significant).

The cytotoxicity of the LDH-P_2_ and LDH-P_5_ conjugates was evaluated using HEK-293T cells, along with all the LDHs from the intermediate steps leading to their synthesis. Figure 11 shows the results at 48 and 72 h for the LDH-P_2_ conjugates (similar results were obtained for LDH-P_5_). With the unmodified LDHs, the relative cell viability remained above 80% across all the evaluated concentrations, confirming their non-toxic nature [53,54], and this is consistent with the findings reported by Govea-Alonso et al. [18] using the same cells and by Wang et al. [19] using 293T cells. A similar behavior was obtained for LDH-NH_2_, except for the highest concentration at 72 h, where low toxicity was noted. At 48 h, LDH-CHO was non-toxic up to 100 µg/mL, while at higher concentrations, it showed low toxicity. By 72 h, the same material at 250 µg/mL was no longer toxic, and low toxicity persisted for the highest concentration, which could be explained by the presence of reactive groups. For the final conjugate (LDH-P_2_), only the highest concentration (500 µg/mL) showed low toxicity. It is worth mentioning that the particle concentration of LDH-P_2_ used for immunogenicity studies remained below 100 µg/mL.

**Figure 12:**
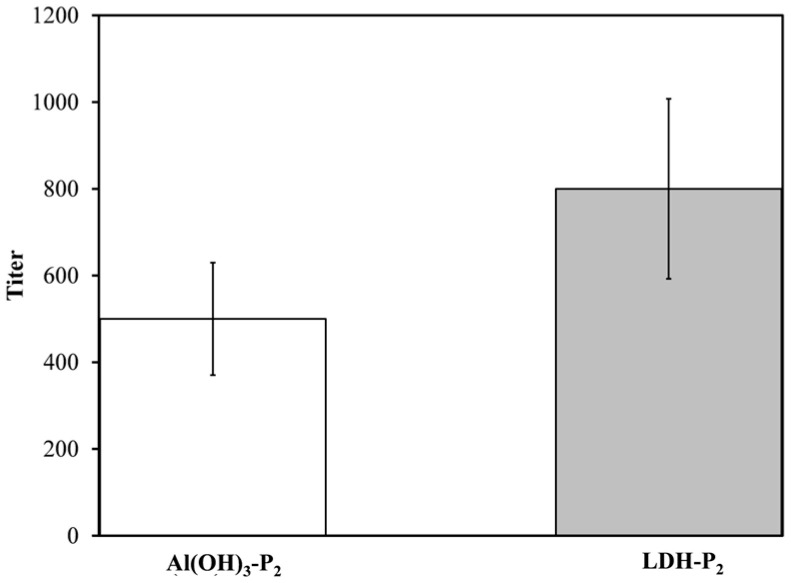
Anti-P_2_ IgG titers induced by immunization with P_2_ bound to LDH or co-administered with alum adjuvant. Anti-P_2_ IgG titers were measured by ELISA using serial dilutions of test sera following booster dose.

Figure 12 shows the immunogenicity results of the LDH-P_2_ nanovaccine measured using anti-P_2_ IgG titers. Interestingly, the LDH-P_2_ nanovaccine induced IgG titers comparable to those induced when alum was used as adjuvant, indicating that LDHs are a potent adjuvant capable of enabling the P_2_ peptide to induce a humoral response. In contrast, the other candidate (LDH-P_5_) did not trigger a detectable IgG response, suggesting that covalent coupling should be evaluated on a case-by-case basis to ensure that the antigenic determinants of the target peptide are preserved during conjugation.

## 4. Conclusions

Layered double hydroxides (LDHs) were synthesized and successfully modified with peptide molecules through covalent conjugation to develop a candidate nanovaccine against SARS-CoV-2. The synthesis process involved silanization, followed by peptide binding using GTA as a linker under reductive amination. Each reaction step was thoroughly characterized to corroborate successful chemical modification. In addition, the reaction conditions were established to minimize their impact on the nanoparticle stability, which allowed us to produce a nanovaccine with a low polydispersity index. The generated subunit nanovaccine (LDH-P_2_) contained a peptide concentration of 125 µg/mL, with an average hydrodynamic size of 428 nm and a PdI of 0.21; moreover, it demonstrated no toxicity at concentrations below 250 µg/mL and exhibited immunogenicity comparable to that of the traditional system that uses aluminum hydroxide as adjuvant. Nevertheless, additional research is needed to enhance reaction efficiency (allowing for reduced peptide usage), decrease the hydrodynamic size of the resulting nanovaccine, and increase the titers of the induced antibodies. This study paves the way to improve the immunogenicity of similar candidates based on peptides chemically bound to the surface of LDHs.

## Figures and Tables

**Table 1:** Final properties of candidate conjugates LDH-P_2_ and LDH-P_5_.

Conjugate	Peptide Concentration (µg/mL)	d_z_ (nm)	PdI
LDH-P_2_	125 ± 16	428 ± 64	0.21 ± 0.10
LDH-P_5_	270 ± 124	252 ± 30	0.14 ± 0.04

**Table 2:** EDS analysis of LDH-NH_2_, LDH-CHO, and LDH-P_2_.

Sample	Element	C	Mg	Al	Si
LDH-NH_2_	Weight%	13.88	18.65	6.34	7.69
	Atomic%	19.87	13.22	4.05	4.71
	%DESV	2.13	1.74	0.50	0.90
LDH-CHO	Weight%	21.62	15.50	6.05	0.21
	Atomic%	29.35	10.44	3.67	0.12
	%DESV	4.14	1.25	0.50	0.07
LDH-P_2_	Weight%	28.60	12.57	6.69	0.16
	Atomic%	37.49	8.14	3.90	0.09
	%DESV	4.26	1.20	0.48	0.03

**Table 3:** Ratio of different elements in LDH, LDH-NH_2_, and LDH-P_2_.

Sample	Mg/Al	Si/LDH	C/LDH	Mg/Si	Mg/C
LDH-NH_2_	3.261	0.273	1.150	2.806	0.665
LDH-CHO	2.842	0.009	2.079	85.590	0.356
LDH-P_2_	2.087	0.007	3.114	90.444	0.217

## Data Availability

The original contributions presented in this study are included in the article. Further inquiries can be directed to the corresponding authors.
